# Comparison of Metabolic Network between Muscle and Intramuscular Adipose Tissues in Hanwoo Beef Cattle Using a Systems Biology Approach

**DOI:** 10.1155/2014/679437

**Published:** 2014-11-13

**Authors:** Hyun-Jeong Lee, Hye-Sun Park, Woonsu Kim, Duhak Yoon, Seongwon Seo

**Affiliations:** ^1^Division of Animal Genomics and Bioinformatics, National Institute of Animal Science, Rural Development Administration, Suwon 441-706, Republic of Korea; ^2^Department of Animal Biosystem Sciences, Chungnam National University, 99 Daehak-ro, Yuseong-gu, Daejeon 305-764, Republic of Korea; ^3^Department of Animal Science, Kyungpook National University, Sangju 742-170, Republic of Korea

## Abstract

The interrelationship between muscle and adipose tissues plays a major role in determining the quality of carcass traits. The objective of this study was to compare metabolic differences between muscle and intramuscular adipose (IMA) tissues in the *longissimus dorsi* (LD) of Hanwoo (*Bos taurus coreanae*) using the RNA-seq technology and a systems biology approach. The LD sections between the 6th and 7th ribs were removed from nine (each of three cows, steers, and bulls) Hanwoo beef cattle (carcass weight of 430.2 ± 40.66 kg) immediately after slaughter. The total mRNA from muscle, IMA, and subcutaneous adipose and omental adipose tissues were isolated and sequenced. The reads that passed quality control were mapped onto the bovine reference genome (build bosTau6), and differentially expressed genes across tissues were identified. The KEGG pathway enrichment tests revealed the opposite direction of metabolic regulation between muscle and IMA. Metabolic gene network analysis clearly indicated that oxidative metabolism was upregulated in muscle and downregulated in IMA. Interestingly, pathways for regulating cell adhesion, structure, and integrity and chemokine signaling pathway were upregulated in IMA and downregulated in muscle. It is thus inferred that IMA may play an important role in the regulation of development and structure of the LD tissues and muscle/adipose communication.

## 1. Introduction

Hanwoo, the dominant breed for beef production in Korea, is known for yielding high quality beef with intense marbling and a high percentage of unsaturated oleic fatty acid [1]. The marbling score is determined by the degree of intramuscular adipose (IMA) deposition in the exposed* longissimus dorsi* (LD) muscle tissue at the 13th rib interface. Consequently, understanding the interrelationship between muscle and adipose tissues in LD is important for improving carcass traits in Hanwoo beef cattle.

Muscle and adipose tissues exhibit distinct metabolism: energy expenditure occurs mainly in muscle while adipose is the main energy storage tissue. Even within adipose tissues, metabolic activities and their regulation are differed by their anatomical locations (e.g., IMA, subcutaneous adipose (SUA), and omental adipose (OMA) depots) [2]. For instance, there are biological differences between SUA and IMA tissues in terms of development, lipid biosynthesis, and substrate preference [3]. In addition, there has been accumulated scientific evidence that adipose and muscle tissues communicate and coregulate each other by secreting various endocrine signal molecules [4–6]. It is reported that the adipose tissue plays a critical role in insulin sensitivity, substrate selection, and oxidative metabolism of skeletal muscle [7, 8]. Little, however, has been studied on differences in metabolic gene network between muscle and IMA in an aspect of deciphering cross communication between these tissues in the LD of beef cattle.

RNA-seq is a powerful tool for profiling and quantifying transcriptome using deep-sequencing technologies [9]. Transcriptome analysis using RNA-seq provides biological insight to understand underlying molecular basis of metabolic differences among different tissues. Recently, the transcriptome expression profile of muscle [10, 11] or adipose [12, 13] tissues in cattle was identified using the RNA-seq technology, and Lee et al. [13] have demonstrated the different depot-specific adipogenesis in Hanwoo. To the best our knowledge, no research has been reported on comparing gene expression profile between muscle and IMA in the LD of beef cattle using RNA-seq.

The objective of this study is thus to compare metabolic differences between muscle and IMA tissues in the LD of Hanwoo on the basis of expression of metabolic gene network using RNA-seq technology and a systems biology approach.

## 2. Materials and Methods

Animal use, care, and experimental protocols for this experiment were reviewed and preapproved by the Institutional Animal Care and Use Committee of the National Institute of Animal Science (number 2010-042).

### 2.1. Animals and Sample Preparation

A total of nine (3 cows, 3 steers, and 3 bulls) Hanwoo cattle (*Bos taurus coreanae*) were used in this study. They were fed the same diet and managed at the same location, Hanwoo Experimental Station in National Institute of Animal Science, throughout the experiment. The average (± standard deviation) carcass weight of the cattle at slaughter was 430.2 (±40.66) kg.

Immediately after slaughter, the LD sections between the 6th and 7th ribs were removed, and the muscle, IMA, and SUA tissues were separated and sampled from this portion. The OMA tissue was taken within the lesser curvature of the abomasum. All of the tissue samples were immediately frozen using liquid nitrogen and stored at −80°C until the analysis.

### 2.2. Total RNA Extraction and RNA-seq Analysis

Total RNA of muscle, IMA, SUA, and OMA tissues were isolated using TRIzol (Invitrogen) and an RNeasy RNA purification kit with DNase treatment (Qiagen). The mRNA was isolated from the total RNA using oligo-dT beads and was reverse transcribed into double strand cDNA fragments. Constructing and sequencing RNA-seq library for each sample were carried out based on protocols of Illumina HiSeq2000 to generate 101 pair-end reads. Quality of RNA-seq reads from all of the tissues was checked using FastQC. The reads that passed the quality control were mapped to Bovine Taurus genome (bosTau6) from UCSC using Tophat2 (v2.0.2) and were counted using HTseq (v0.5.3p3). The gene models and annotations of the bovine genome were obtained from the Ensembl release 72 [14]. The RNA sequencing data from this study have been submitted to the NCBI Gene Expression Omnibus (GEO) under the accession number of GSE39618 (http://www.ncbi.nlm.nih.gov/geo/).

### 2.3. Statistical and Network Analysis

Using the DESeq package in* R* and Bioconductor differentially expressed genes (DEG) across various tissues were identified by analyzing the mapped RNA sequencing reads [15]. In the DEG analysis a negative binomial model was used for normalization, and a generalized linear model was fitted with sex of the cattle as a block in order to account for the variations from the differences in sex. The Bonferroni test with an experimental-wise alpha level of 0.05 was used to identify the list of genes differentially expressed among the tissues. The hierarchical clustering and principle component analysis clustering analyses on DEG were conducted and visualized using the DESeq package [15].

Among the DEG across the tissues, tissue-specific oppositely regulated genes (ORG) were identified by pairwise comparisons of the expression of each gene among different tissues. The criterion used in this study was the sign of log2 of the fold change which indicates a relative level of expression of each gene between two tissues. If the sign of log2 of the fold change of a gene was different in a tissue compared to the other three tissues, the gene was regarded as a tissue-specific ORG. Consequently muscle-specific up- or downregulated genes were relatively less or more expressed, respectively, in all of the adipose tissues. Likewise, intramuscular adipose-specific up- or downregulated genes were relatively less or more expressed, respectively, in muscle and the other adipose tissues.

Functional annotations and enrichment tests of tissue-specific ORG were carried out using the Database for Annotation, Visualization and Integrated Discovery (DAVID) [16]. The significantly enriched KEGG pathways in each of the four different gene sets (upregulated in IMA, downregulated in IMA, upregulated in MUS, and downregulated in MUS) were separately identified against the annotated bovine genome using the Bonferroni test at *P* < 0.05.

## 3. Results

### 3.1. Identification of Oppositely Regulated Genes (ORG)

Totals of 34.2, 35.8, 35.1, and 38.1 Mb of raw reads were obtained on average from muscle, IMA, SUA, and OMA tissues, respectively. More than 99.5% of the reads were remained after the quality filtering passed through the quality control, and more than 95.9% of these were mapped to the reference genome (Supplementary Table 1; see Supplementary Material available online at http://dx.doi.org/10.1155/2014/679437).

**Figure 1:**
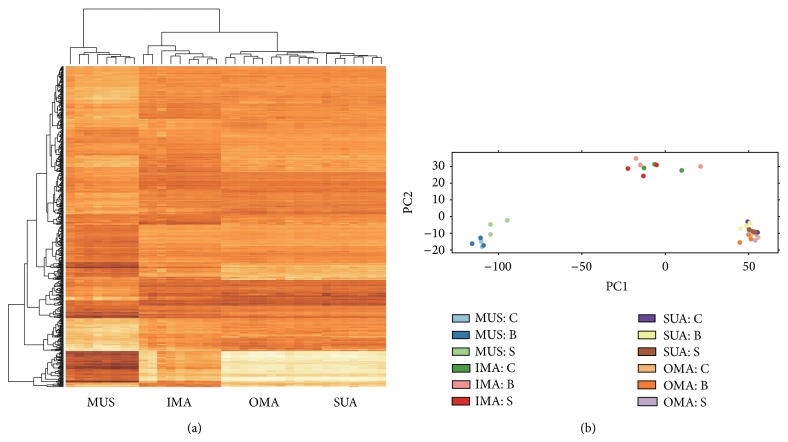
Transcriptomic profiles and clustering of differentially expressed genes (DEG) among four different tissues and three sexes in Hanwoo. The tissues were muscle (MUS), intramuscular adipose (IMA), subcutaneous adipose (SUA), and omental adipose (OMA), and the sexes were cow (C), bull (B), and steer (S). (a) Heat map of levels of gene expressions and hierarchical clustering and (b) clustering using principle component analysis of DEG.

The DEG analysis identified that a total of 7,282 genes were differentially expressed among muscle, IMA, SUA, and OMA tissues. The hierarchical clustering analysis on the expressions of DEG clearly showed distinct patterns of gene expression between muscle and adipose tissues and a unique gene expression profile of IMA compared to OMA and SUA (Figure 1(a)). This was also confirmed by a clustering analysis using principle component analysis (Figure 1(b)). Regardless of sexes, SUA and OMA were clustered together, and there were the other two distinct clusters: muscle and IMA.

The total number of muscle tissue-specific ORG was 6,385 genes in which 2,803 genes were upregulated while 3,582 genes were downregulated. On the other hand, a relatively fewer number of genes (3,113) were differentially expressed in intramuscular adipose tissue: 1,970 genes were upregulated while 1,143 were downregulated.

The KEGG pathway enrichment analysis on each of the four sets of genes (upregulated in IMA, downregulated in IMA, upregulated in MUS, and downregulated in MUS) revealed the opposite direction of metabolic regulation between MUS and IMA (Tables 1 and 2). Among nine significantly upregulated KEGG pathways in the IMA, five pathways (i.e., focal adhesion, ECM-receptor interaction, axon guidance, chemokine signaling pathway, and regulation of actin cytoskeleton) were downregulated in the muscle tissue. On the other hand, among 11 downregulated pathways in the IMA, seven pathways (i.e., oxidative phosphorylation Parkinson's disease, Huntington's disease, Alzheimer's disease, citric acid cycle, proteasome, and pyruvate metabolism) were upregulated in muscle (Table 1). In addition, valine, leucine, and isoleucine degradation, propanoate metabolism, fatty acid degradation, and butanoate metabolism were downregulated in the intramuscular adipose tissue. Seven out of 13 upregulated pathways in the muscle tissue were downregulated in the intramuscular adipose tissues, while five out of 12 downregulated pathways in muscle were upregulated in intramuscular adipose (Table 2). Interestingly, it was indicated that the KEGG pathway of dilated cardiomyopathy was upregulated in both IMA and MUS although different ORG was identified.

Metabolic gene network analysis clearly indicated that oxidative metabolism, such as oxidative phosphorylation, citric acid cycle, pyruvate metabolism, glycolysis, and purine metabolism, was upregulated in the muscle tissue but downregulated in the intramuscular tissue. Oxidation of branch-chained amino acids and short-chain fatty acids was specifically downregulated in the intramuscular tissue compared to muscle and subcutaneous adipose and omental adipose tissues.

Interestingly enough, pathways involved in regulation of cell adhesion, structure, and integrity (i.e., ECM adhesion, ECM-receptor interaction, axon guidance, and regulation of actin cytoskeleton) and chemokine signaling pathway were upregulated in the intramuscular adipose tissue and downregulated in the muscle tissue. It is thus speculated that intramuscular adipose tissue may play an important role in regulation of development and structure of the LD tissues and muscle and adipose communication.

## 4. Discussion

Carcass traits are influenced by various factors including genotype, age, nutrition, and management. The IMA and back fat thickness are increased by age [17] and time on feed [18]. The back fat thickness increases more rapidly than marbling score by time on feed. Therefore, extending the feeding period to improve carcass traits may be counterproductive [18]. The IMA develops later on the course of growing and behaves differently from other types of adipose (e.g., subcutaneous and omental adipose tissues) in terms of cellularity and metabolic characteristics [2], and it secretes signals and cross talks with the muscle tissue [4]. Thus, we need to find a way to increase the intramuscular adipose content without deposition of other types of adipose tissues. Better understanding of the interrelationship between intramuscular adipose and muscle tissues in LD is a prerequisite.

The present study identified a large number (7,281) of DEG among muscle, IMA, SUA, and OMA tissues in Hanwoo using RNA-seq. The DEG obtained using the RNA-seq analysis were not fully cross validated by qRT-PCR in this study although some of the genes showed a significant and strong correlation between RNA-seq and qRT-PCR previously. In our previous analysis with the same RNA samples, the expression levels of ten randomly selected genes each from IMA, SUA, and OMA are higher than 0.85 of Pearson's correlation coefficient [13].

Clustering analysis of DEG showed that each tissue exhibited unique gene expression pattern regardless of genders (i.e., cow, steer, or bull) or biological replications even though gene expression profiles of SUA and OMA tissues were similar. On the basis of different gene expression pattern between muscle and IMA, it is clear that IMA was successfully isolated from LD in this study in spite of its experimental difficulties. Relative to muscle, the adipose tissues clustered together, and IMA showed different pattern from OMA and SUA tissues. Sheng et al. [12] reported a total of 953, 1,534, and 2,026 DEG between IMA and SUA, IMA and perirenal adipose, and SUA and perirenal adipose tissues, respectively, and found 323 DEG in IMA compared to external adipose tissues. Transcriptome studies in other species have also reported that gene expression patterns were clustered by the tissue rather than sex or breed types [19–21].

We identified not only differentially expressed but also muscle- or intramuscular adipose-specific oppositely (up- versus down)regulated genes. A relatively larger number of genes were oppositely regulated in the muscle tissue compared to the others. Metabolic pathway enrichment analysis with the ORG in muscle and IMA revealed the opposite direction of metabolic regulation between muscle and IMA: oxidative pathways were upregulated in muscle but downregulated in intramuscular adipose. Pyruvate metabolism, citric acid cycle, and oxidative phosphorylation were upregulated in muscle. Particularly the genes encoding two core enzymes of pyruvate metabolism, lactate dehydrogenase, and pyruvate dehydrogenase complex were oppositely upregulated in muscle compared to adipose tissues. Pyruvate metabolism is known to play a pivotal role in aerobic and anaerobic energy metabolism. When oxygen is sufficient, pyruvate is oxidized to acetyl-CoA by pyruvate dehydrogenase. Acetyl-CoA then enters into citric acid cycle, and oxidative phosphorylation generates ATP. On the other hand, during intense muscular activity, pyruvate is converted to lactate by lactate dehydrogenase anaerobically [22, 23].

The pathways involved in regulation of cell adhesion, structure, and integrity and chemokine signaling pathway were upregulated in intramuscular adipose but downregulated in muscle. Specifically ECM adhesion and ECM-receptor interaction pathways are critical in these functions. These pathways have also been proposed to play an important role in the intramuscular fat deposition in chicken [24]. The ECM surrounds cells and accomplishes a number of specific functions, such as cell adhesion, migration, proliferation, and differentiation. In muscle, cells are tightly bound together, and the extracellular spaces containing the ECM are limited. The ECM is mainly composed of two macromolecules: glycosaminoglycans and fibrous proteins. Glycosaminoglycans are usually covalently linked to core proteins to form proteoglycans, and fibrous proteins can be divided into two functional groups: structural (e.g., collagen, elastin) and adhesive (e.g., fibronectin, laminin, and vitronectin) types [25]. Transmembrane molecules such as integrins mediate interactions between cells and the ECM. In our study, collagen, integrin, laminin, fibronectin, and other cell junction related genes were significantly upregulated in IMA. Interestingly enough, some of the upregulated pathways in the IMA tissue were highly expressed in the LD of cattle embryo. He and Liu [11] identified DEG in the LD muscle between embryo and 30-month-old adult cattle using RNA-seq and conducted KEGG pathway enrichment analysis. They reported that KEGG pathways of axon guidance, hypertrophic cardiomyopathy, dilated cardiomyopathy, pathways in cancer, and regulation of actin cytoskeleton were significantly and highly expressed in embryo compared to adult.

To the best of our knowledge, this study is the first scientific report suggesting that intramuscular adipose tissue may play an important role in communication with muscle and regulation of development and structural integrity of the* longissimus dorsi* tissue. The findings from this study will provide a scientific ground for finding better strategies to improve carcass traits of beef cattle.

Moreover, this study used the RNA-seq technology and a systems biology approach, which are relatively new to our research field but powerful tools for quantifying and profiling transcriptome (the whole set of expressed genes) and deciphering underlying molecular basis of metabolic differences among different tissues. Application of these recent techniques in the animal science field should be very helpful for expanding our understanding of animal biology.

## Supplementary Material

The average number of raw reads in muscle, IMA, SUA, and OMA tissues were 34.2, 35.8, 35.1, and 38.1 Mb, respectively. Among the raw reads passed the quality control, and more than 95.9% were mapped to the reference genome.

## Figures and Tables

**Table 1:** List of oppositely up- or downregulated KEGG pathways in the *longissimus dorsi* intramuscular adipose tissue compared to muscle and subcutaneous adipose and omental adipose tissues in Hanwoo.

	KEGG ID	Pathway	Fold enrichment	Bonferroni
Upregulated	bta04510	Focal adhesion^†^	2.7	7.0*E* − 09
	bta04512	ECM-receptor interaction^†^	3.7	8.2*E* − 08
	bta04360	Axon guidance^†^	2.8	1.5*E* − 05
	bta05200	Pathways in cancer	1.8	1.6*E* − 03
	bta04270	Vascular smooth muscle contraction	2.4	3.7*E* − 03
	bta04062	Chemokine signaling pathway^†^	2.0	9.9*E* − 03
	bta05412	Arrhythmogenic right ventricular cardiomyopathy	2.8	1.3*E* − 02
	bta04810	Regulation of actin cytoskeleton^†^	1.9	1.9*E* − 02
	bta05414	Dilated cardiomyopathy	2.5	4.2*E* − 02

Downregulated	bta00190	Oxidative phosphorylation^‡^	6.2	2.4*E* − 23
	bta05012	Parkinson's disease^‡^	6.0	7.9*E* − 21
	bta05016	Huntington's disease^‡^	4.8	7.5*E* − 18
	bta05010	Alzheimer's disease^‡^	4.8	8.4*E* − 17
	bta00020	Citrate cycle (TCA cycle)^‡^	10.5	4.9*E* − 13
	bta03050	Proteasome^‡^	7.0	1.4*E* − 08
	bta00280	Valine, leucine, and isoleucine degradation	5.9	1.2*E* − 05
	bta00640	Propanoate metabolism	6.7	8.8*E* − 05
	bta00071	Fatty acid degradation	5.4	8.7*E* − 04
	bta00650	Butanoate metabolism	5.4	8.1*E* − 03
	bta00620	Pyruvate metabolism^‡^	4.7	2.7*E* − 02

^†^Downregulated in the muscle tissue.

^‡^Upregulated in the muscle tissue.

**Table 2:** List of oppositely up- or downregulated KEGG pathways in the *longissimus dorsi* muscle tissue compared to intramuscular, subcutaneous, and omental adipose tissues in Hanwoo.

	KEGG ID	Pathway	Fold enrichment	Bonferroni
Upregulated	bta05012	Parkinson's disease^†^	4.2	1.4*E* − 27
	bta05010	Alzheimer's disease^†^	3.7	5.3*E* − 26
	bta00190	Oxidative phosphorylation^†^	4.0	8.3*E* − 26
	bta05016	Huntington's disease^†^	3.4	2.2*E* − 22
	bta03050	Proteasome^†^	5.3	9.5*E* − 15
	bta04260	Cardiac muscle contraction	3.7	3.2*E* − 10
	bta00020	Citrate cycle (TCA cycle)^†^	5.1	8.7*E* − 09
	bta05410	Hypertrophic cardiomyopathy (HCM)	2.7	4.9*E* − 04
	bta05414	Dilated cardiomyopathy	2.5	1.9*E* − 03
	bta00230	Purine metabolism	2.0	2.8*E* − 03
	bta04120	Ubiquitin mediated proteolysis	2.1	3.2*E* − 03
	bta00620	Pyruvate metabolism^†^	3.0	2.7*E* − 02
	bta00010	Glycolysis/gluconeogenesis	2.6	2.9*E* − 02

Downregulated	bta04510	Focal adhesion^‡^	1.9	1.3*E* − 06
	bta04512	ECM-receptor interaction^‡^	2.4	5.0*E* − 06
	bta04640	Hematopoietic cell lineage	2.3	1.9*E* − 05
	bta04062	Chemokine signaling pathway^‡^	1.8	4.7*E* − 05
	bta04360	Axon guidance^‡^	2.0	7.5*E* − 05
	bta04670	Leukocyte transendothelial migration	2.0	1.1*E* − 04
	bta04060	Cytokine-cytokine receptor interaction	1.7	6.3*E* − 04
	bta00600	Sphingolipid metabolism	2.7	3.7*E* − 03
	bta04514	Cell adhesion molecules (CAMs)	1.8	4.2*E* − 03
	bta04810	Regulation of actin cytoskeleton^‡^	1.6	5.9*E* − 03
	bta04142	Lysosome	1.8	6.6*E* − 03
	bta04666	Fc gamma R-mediated phagocytosis	2.0	8.6*E* − 03

^†^Downregulated in the intramuscular adipose tissue.

^‡^Upregulated in the intramuscular adipose tissue.
