# Supplementary material for: Comparison of Metabolic Network between Muscle and Intramuscular Adipose Tissues in Hanwoo Beef Cattle Using a Systems Biology Approach
Source: Int J Genomics. 2014 Nov 13;2014:679437. doi: 10.1155/2014/679437 (PMC4247929; doi:10.1155/2014/679437)
Supplement: Supplementary file 1 — The average number of raw reads in muscle, IMA, SUA, and OMA tissues were 34.2, 35.8, 35.1, and 38.1 Mb, respectively. Among the raw reads passed the quality control, and more than 95.9% were mapped to the reference genome. [file 679437.f1.pdf]

Supplementary Table 1. Summary of RNA-seq data from four different tissues (MUS: muscle, IMA: intramuscular adipose, SUA: subcutaneous adipose, and OMA: omental adipose) and three sexes of Hanwoo.

Animal	Sex	Tissue	Raw reads	Reads after QC	Mapped reads	Mapped rate (%)
27258	Bull	IMA	32,795,970	32,791,028	32,079,358	97.8
		MUA	33,998,572	33,907,725	32,510,294	95.9
		OMA	40,145,136	40,139,167	39,471,224	98.3
		SUA	34,961,934	34,956,668	33,887,004	96.9
28298	Bull	IMA	25,404,790	25,379,357	25,094,287	98.9
		MUA	41,028,336	40,928,050	40,196,483	98.2
		OMA	33,774,810	33,741,132	32,878,392	97.4
		SUA	31,265,648	31,234,834	30,588,262	97.9
28300	Bull	IMA	33,896,998	33,887,341	33,013,485	97.4
		MUA	37,633,852	37,529,831	36,397,717	97.0
		OMA	37,094,812	37,083,976	35,835,326	96.6
		SUA	36,409,346	36,398,844	35,037,306	96.3
28046	Cow	IMA	38,957,018	38,917,997	38,194,374	98.1
		MUA	-		-	-
		OMA	41,075,552	41,034,551	40,914,740	99.7
		SUA	37,902,974	37,865,228	36,992,627	97.7
28049	Cow	IMA	27,859,998	27,852,084	26,789,222	96.2
		MUA	25,385,914	25,308,868	24,358,982	96.3
		OMA	36,559,594	36,549,104	35,924,487	98.3
		SUA	31,844,692	31,835,650	30,785,146	96.7
28057	Cow	IMA	38,983,416	38,977,599	38,573,994	99.0
		MUA	32,232,094	32,110,013	30,957,247	96.4
		OMA	49,285,572	49,278,117	47,590,598	96.6
		SUA	29,063,304	29,058,910	28,119,540	96.8
28260	Steer	IMA	42,173,502	42,161,496	41,159,915	97.6
		MUA	38,415,258	38,290,257	36,857,769	96.3
		OMA	37,910,536	37,899,902	36,510,193	96.3
		SUA	36,503,342	36,492,884	35,224,619	96.5
28261	Steer	IMA	36,733,112	36,696,313	36,183,925	98.6
		MUA	28,173,380	28,097,819	27,304,816	97.2
		OMA	33,236,374	33,203,354	32,295,059	97.3
		SUA	37,683,440	37,645,749	36,578,016	97.2
28271	Steer	IMA	45,547,786	45,540,853	45,472,436	99.9
		MUA	37,007,276	36,811,521	35,816,734	97.3
		OMA	33,840,910	33,835,854	32,628,247	96.4
		SUA	40,603,752	40,597,681	39,131,638	96.4
